# Liproxstatin-1 Protects SH-SY5Y Cells by Inhibiting H_2_O_2_-Induced Excessive Mitophagy and Apoptosis

**DOI:** 10.3390/ijms262311641

**Published:** 2025-12-01

**Authors:** Tingting Yan, Feng Ding, Zhongyuan Fang, Yan Zhao

**Affiliations:** Department of Bioengineering, Harbin Institute of Technology, Weihai 264209, China

**Keywords:** liproxstatin-1, mitophagy, apoptosis, AKT/mTOR, H_2_O_2_

## Abstract

Oxidative stress is a critical factor in the pathogenesis of various neuronal disorders, causing cellular damage and mitochondrial dysfunction. This study aimed to explore the protective effects of liproxstatin-1 against H_2_O_2_-induced neural oxidative damage and elucidate the underlying mechanisms. Our findings demonstrated that 500 μmol/L H_2_O_2_ treatment induced mitochondrial dysfunction and apoptosis in SH-SY5Y cells, while 1 μmol/L liproxstatin-1 effectively mitigated these cytotoxic effects by restoring mitochondrial integrity and enhancing cell viability. Furthermore, 500 μmol/L H_2_O_2_ exposure significantly suppressed the activation of the protein kinase B/ mammalian target of rapamycin signaling pathway and triggered excessive mitophagy. Pretreatment with 1 μmol/L liproxstatin-1 attenuated the damage by H_2_O_2_, suggesting its protective role. Collectively, our results indicated that 500 μmol/L H_2_O_2_ induces cytotoxicity through oxidative damage, protein kinase B/ mammalian target of rapamycin pathway inhibition, and aberrant mitophagy, ultimately leading to apoptosis; meanwhile, 1 μmol/L liproxstatin-1 counteracted these effects by preserving mitochondrial function, suppressing excessive mitophagy, and inhibiting apoptotic pathways, thereby protecting SH-SY5Y cells from H_2_O_2_-induced cytotoxicity.

## 1. Introduction

Mitochondria play a pivotal role in determining cell fate by regulating the balance between cell survival and death [1]. Under physiological conditions, mitochondria maintain cellular homeostasis through energy production and redox regulation. Oxidative stress exacerbates mitochondrial injury, inducing excessive mitophagy, a selective form of autophagy that degrades damaged mitochondria [2]. Enhanced mitophagy has been implicated in promoting cell death through the activation of proapoptotic pathways [1]. Excessive mitophagy can induce apoptosis, particularly via Parkin overexpression or by augmenting Parkin activity through the inhibition of myeloid cell leukemia-1, a mitochondrial-specific deubiquitylase [3]. The two main apoptotic pathways are the extrinsic pathway and the intrinsic pathway, the latter of which is mediated by mitochondria. In the intrinsic pathway, Bcl-2 family proteins mediate the opening of the mitochondrial permeability transition pore, leading to the release of cytochrome c from the mitochondria into the cytosol, thereby amplifying caspase activity [4]. The protein kinase B (Akt)/mammalian target of rapamycin (mTOR) signaling axis plays a critical role in modulating both mitophagy and cell survival. Activation of the Akt/mTOR pathway suppresses apoptosis by inhibiting mitophagy [5]. Conversely, downregulation of Akt/mTOR signaling enhances mitophagy-dependent apoptosis, which may contribute to neuronal degeneration and cognitive impairment [6].

Antioxidant therapy has emerged as a promising strategy to mitigate the overproduction of reactive oxygen species (ROS) by damaged mitochondria, alleviate oxidative stress, and restore mitochondrial function, thereby preventing excessive mitophagy and apoptosis [7], holding significant therapeutic potential for neurodegenerative diseases. SH-SY5Y cells, a well-characterized human neuroblastoma cell line, share phenotypic and functional similarities with primary neurons [6]. These properties make them a widely used, reliable in vitro model for studying neurodegenerative disease pathogenesis and evaluating neuroprotective agents. Liproxstatin-1, a well-recognized ferroptosis inhibitor, has been shown to alleviate steatosis and steatohepatitis by blocking PANoptosis [8], and it also exerts protective effects against oxidative stress, lipid peroxidation, mitochondrial injury, and neuronal damage [9,10]. However, the specific mechanisms by which liproxstatin-1 protects neuronal cells from H_2_O_2_-induced damage, particularly its regulatory role in excessive mitophagy and apoptosis, which are key pathological events in oxidative stress-related neuronal injury, remain unclear.

To fill this research gap, the present study aimed to: (1) establish an H_2_O_2_-induced oxidative stress model in SH-SY5Y cells to mimic mitochondrial dysfunction in neurodegenerative diseases; (2) investigate the protective effect of liproxstatin-1 against H_2_O_2_-induced cytotoxicity; and (3) explore whether its protective mechanism involves the regulation of the Akt/mTOR signaling pathway, as well as the inhibition of excessive mitophagy and apoptosis. The findings of this study are expected to provide novel insights into the potential application of liproxstatin-1 in the treatment of neurodegenerative diseases associated with oxidative stress.

## 2. Results

### 2.1. Liproxstatin-1 Preserves Cell Viability and Mitochondrial Function in H_2_O_2_-Exposed SH-SY5Y Cells

**Figure 1:**
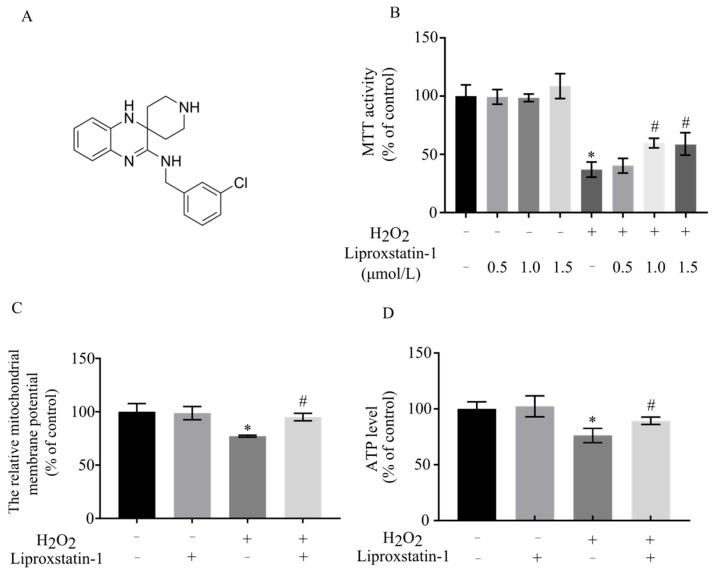
Liproxstatin-1 preserves cell viability and mitochondrial function in H_2_O_2_-exposed SH-SY5Y cells. (**A**) The chemical structure of liproxstatin-1. (**B**) SH-SY5Y cells were pretreated with 0.5, 1, and 1.5 μmol/L liproxstatin-1 for 24 h, followed by exposure to 500 μmol/L H_2_O_2_ for 2 h. MTT assay was then performed. SH-SY5Y cells were pretreated with 1 μmol/L liproxstatin-1 for 24 h, followed by exposure to 500 μmol/L H_2_O_2_ for 2 h. (**C**) mitochondrial membrane potential assay, and (**D**) ATP assay were then performed. Data are represented as mean ± SD of 3 independent experiments. * *p* < 0.05 versus control, # *p* < 0.05 versus H_2_O_2_-treated cells.

Initially, H_2_O_2_ was used to induce oxidative stress in vitro, and the cytotoxic effects on SH-SY5Y cells were evaluated. Figure 1A presents the chemical structure of liproxstatin-1, highlighting its key functional moieties (pyrazole core and hydrophobic alkyl chain) that are critical for its ferroptosis inhibitory and neuroprotective activities [11]. As shown in Figure 1B, the viability of SH-SY5Y cells exposed to H_2_O_2_ decreased to approximately 30% of the control group, confirming the successful establishment of the oxidative stress damage model. In our prior study, astaxanthin (optimal concentration: 80 μg/L) significantly protected SH-SY5Y cells against H_2_O_2_-induced damage, as evidenced by restored cell viability, mitochondrial function, and inhibited apoptosis, which serves as a robust benchmark for evaluating novel neuroprotective agents in this experimental model [12].

In the current study, cells were pretreated with 0.5, 1, and 1.5 μmol/L liproxstatin-1 before H_2_O_2_ treatment. As shown in Figure 1B, liproxstatin-1 pretreatment dose-dependently improved cell survival, with cell viability rates increasing to approximately 55% and 53% at 1 μmol/L and 1.5 μmol/L liproxstatin-1, respectively, indicating significant protective effects against H_2_O_2_-induced cellular damage. Notably, the protective magnitude of 1 μmol/L liproxstatin-1 was comparable to that of astaxanthin in our published study, validating the biological relevance activity of liproxstatin-1 [12]. Given its optimal protective efficacy and consistency with the reference performance of astaxanthin in our previous study [12], 1 μmol/L liproxstatin-1 was selected for subsequent experiments.

A decrease in mitochondrial membrane potential is a key indicator of mitochondrial dysfunction [13]. As shown in Figure 1C, treatment with H_2_O_2_ decreased the mitochondrial membrane potential to 77% of the control group, while this depolarization was significantly suppressed by 1 μmol/L liproxstatin-1 pretreatment. Similarly, liproxstatin-1 treatment abolished the detrimental effect of H_2_O_2_ on ATP level (Figure 1D), a critical parameter reflecting mitochondrial metabolic function. These protective effects of liproxstatin-1 on mitochondrial integrity and energy metabolism are consistent with the established role of astaxanthin in our prior work, further confirming that liproxstatin-1 exerts specific cytoprotection against H_2_O_2_-induced oxidative damage.

### 2.2. Liproxstatin-1 Attenuates Oxidative Stress in H_2_O_2_-Treated SH-SY5Y Cells

**Figure 2:**
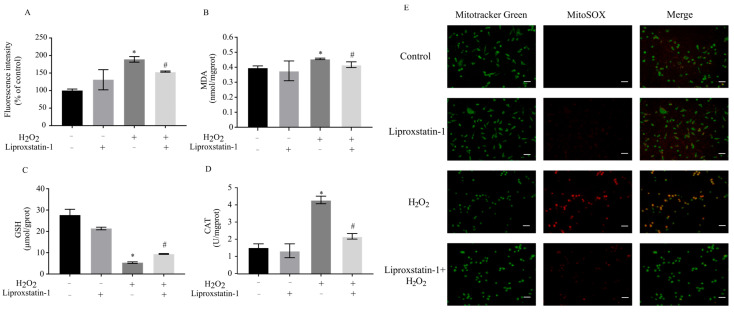
Effects of liproxstatin-1 on oxidative stress in H_2_O_2_-treated SH-SY5Y cells. SH-SY5Y cells were pretreated with 1 μmol/L liproxstatin-1 for 24 h, followed by exposure to 500 μmol/L H_2_O_2_ for 2 h. (**A**) Intracellular ROS, (**B**) MDA, (**C**) GSH, (**D**) CAT detection, and (**E**) Mitochondrial ROS assays were then performed. Scale bar, 50 μm. Data are presented as mean ± SD of 3 independent experiments. * *p* < 0.05 versus control, # *p* < 0.05 versus H_2_O_2_-treated cells.

Liproxstatin-1, a potent inhibitor of lipid autoxidation, has been demonstrated to reduce ROS, 4-HNE, and MDA levels while upregulating GSH in renal tubular epithelial cells following 2-deoxy-d-ribose treatment [14]. To further investigate its antioxidant properties, we examined the effects of liproxstatin-1 in H_2_O_2_-treated SH-SY5Y cells. As shown in Figure 2A,E, H_2_O_2_ treatment increased intracellular ROS levels to 1.89-fold compared to the control group and elevated mitochondrial ROS, whereas liproxstatin-1 pretreatment significantly attenuated these effects. A similar trend was observed in MDA levels (Figure 2B). Additionally, H_2_O_2_ exposure markedly decreased GSH content, but liproxstatin-1 pretreatment restored GSH to significantly higher levels (Figure 2C). In contrast, CAT activity was significantly elevated in H_2_O_2_-treated cells. However, liproxstatin-1 pretreatment substantially suppressed this H_2_O_2_-induced increase (Figure 2D).

### 2.3. Liproxstatin-1 Modulates Mitophagy and Mitophagy-Related Protein Expression in H_2_O_2_-Treated SH-SY5Y Cells

**Figure 3:**
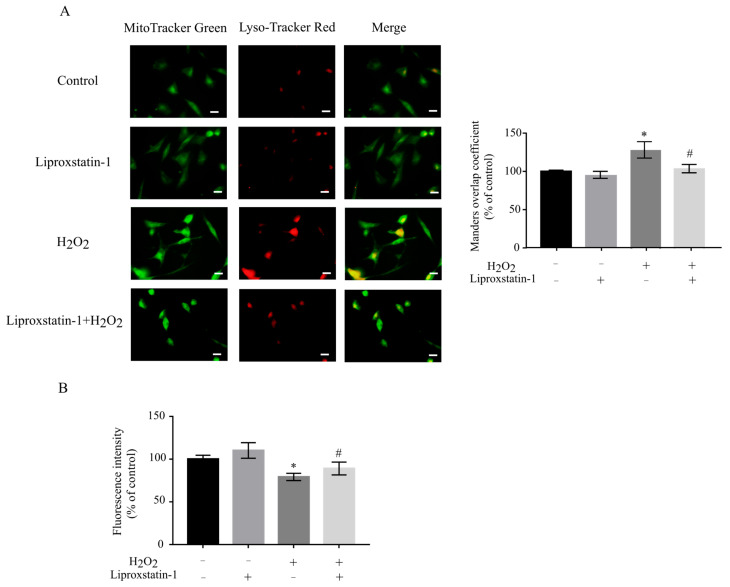
Effects of liproxstatin-1 on mitophagy in H_2_O_2_-treated SH-SY5Y cells. SH-SY5Y cells were pretreated with 1 μmol/L liproxstatin-1 for 24 h, followed by exposure to 500 μmol/L H_2_O_2_ for 2 h. (**A**) Co-localization of MitoTracker Green and Lyso-Tracker Red. The histograms show values of the Manders overlap coefficient of fluorescence intensity. Scale bar, 10 μm. (**B**) The NAO staining assay was performed. Data are presented as mean ± SD of 3 independent experiments. * *p* < 0.05 versus control, # *p* < 0.05 versus H_2_O_2_-treated cells.

Mitophagy serves as a crucial mitochondrial quality control mechanism under oxidative stress conditions. While oxidative stress hyperactivates mitophagy, it concurrently induces mitochondrial dysfunction [15]. Mitochondria and lysosomes were specifically labeled using MitoTracker Green and LysoTracker Red fluorescent probes, respectively, while mitophagy activity was subsequently quantified by analyzing the co-localization coefficient of these fluorescent signals [16]. Previous research has demonstrated that liproxstatin-1 significantly influences key signaling pathways governing cell proliferation and death, particularly those regulating autophagy and apoptosis [17]. Our findings reveal that H_2_O_2_ treatment elevated the Manders overlap coefficient to approximately 120% (Figure 3A), while combined liproxstatin-1 and H_2_O_2_ treatment resulted in a lower increase (about 110%). These data suggest that liproxstatin-1 exerts a protective effect against H_2_O_2_-induced autophagic dysregulation. Furthermore, the mitochondrial mass was measured by the fluorescent dye NAO [18]. Quantitative analysis using the fluorescent dye NAO demonstrated that liproxstatin-1 effectively attenuated the H_2_O_2_-mediated reduction in mitochondrial mass (Figure 3B).

**Figure 4:**
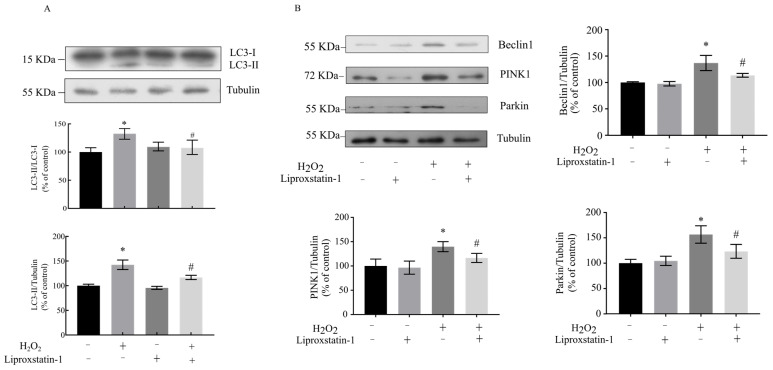
Liproxstatin-1 modulates mitophagy-related protein expression in H_2_O_2_-treated SH-SY5Y cells. SH-SY5Y cells were pretreated with 1 μmol/L liproxstatin-1 for 24 h, followed by exposure to 500 μmol/L H_2_O_2_ for 2 h. (**A**,**B**) Protein levels of LC3, Beclin1, PINK1, Parkin, and Tubulin in whole cell lysates as determined by Western blot analyses. The band intensities were quantified by densitometric analyses and normalized according to the amount of Tubulin. Data are presented as mean ± SD of 3 independent experiments. * *p* < 0.05 versus control, # *p* < 0.05 versus H_2_O_2_-treated cells.

To further investigate whether liproxstatin-1 modulates mitophagy in the context of H_2_O_2_-induced oxidative stress, we evaluated the expression of key mitophagy and autophagy markers via Western blot analysis (Figure 4). Mitophagy is tightly regulated by the PINK1/Parkin pathway (a core mediator of mitochondrial quality control) and canonical autophagy-related proteins (e.g., Beclin1, LC3), whose expression levels directly reflect mitophagic activity.

Beclin1 serves as a critical regulator in the initiation of autophagosome membrane formation [19]. During mammalian autophagy activation, LC3-I (the processed form of LC3) undergoes further cleavage to generate LC3-II, whose accumulation serves as a reliable indicator of autophagic activity [20]. Notably, high-dose liproxstatin-1 exhibits functional similarities to established lysosomal inhibitors (chloroquine and bafilomycin A1), including the expansion of acidic vesicle compartments and upregulation of autophagy-lysosomal pathway-associated proteins [21]. Our experimental results demonstrate that H_2_O_2_ treatment elevated the ratios of LC3-II/LC3-I, LC3-II/Tubulin, and Beclin1/Tubulin to approximately 140%, 140%, and 136%, respectively (Figure 4A,B). However, liproxstatin-1 pretreatment effectively attenuated these H_2_O_2_-induced increases.

Parkin represents a central mediator of mitophagy initiation, facilitating ubiquitination of mitochondrial outer membrane proteins and subsequent degradation of impaired mitochondria. The coordinated action of Parkin and PINK1 constitutes a fundamental mitophagy pathway in neuronal cells [22]. As shown in Figure 4B, H_2_O_2_ exposure increased PINK1/Tubulin and Parkin/Tubulin levels to approximately 130% and 160%, respectively. Importantly, liproxstatin-1 pretreatment significantly reduced these H_2_O_2_-induced elevations in both PINK1 and Parkin expression. These findings collectively indicate that liproxstatin-1 potently suppresses H_2_O_2_-triggered mitophagy activation.

### 2.4. Liproxstatin-1 Attenuates H_2_O_2_-Induced Apoptosis in SH-SY5Y Cells via Regulation of Mitochondrial Pathways

**Figure 5:**
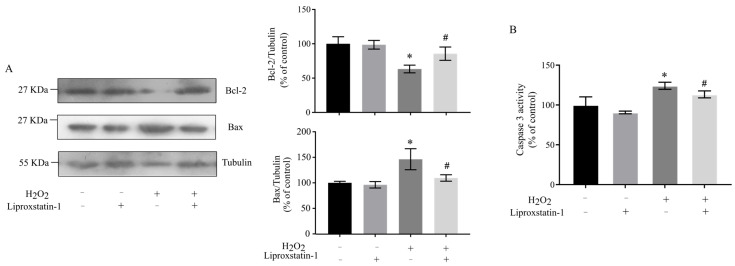
Liproxstatin-1 attenuates H_2_O_2_-induced apoptosis in SH-SY5Y cells. SH-SY5Y cells were pretreated with 1 μmol/L liproxstatin-1 for 24 h, followed by exposure to 500 μmol/L H_2_O_2_ for 2 h. (**A**) Protein levels of Bcl-2, Bax, and Tubulin in whole cell lysates as determined by Western blot analyses. The band intensities were quantified by densitometric analyses and normalized according to the amount of Tubulin. (**B**) The caspase 3 activities were determined. Data are presented as mean ± SD of 3 independent experiments. * *p* < 0.05 versus control, # *p* < 0.05 versus H_2_O_2_-treated cells.

As highly dynamic and multifunctional organelles, mitochondria play a central role in regulating cellular survival and death, with their dysfunction being closely associated with various diseases, particularly neurodegenerative disorders [2]. The Bcl-2 protein family, consisting of pro-apoptotic members like Bax and anti-apoptotic members like Bcl-2, serves as a critical regulator of mitochondrial-mediated apoptosis, where Bcl-2 exerts its protective effects by antagonizing Bax activity through membrane integration [23]. Our investigation into the protective mechanism of liproxstatin-1 against H_2_O_2_-induced apoptosis revealed significant alterations in key apoptotic markers. Specifically, H_2_O_2_ treatment led to a 30% reduction in Bcl-2 protein levels and a marked upregulation of both Bax expression and caspase 3 activity (increased by approximately 130%), while liproxstatin-1 pretreatment effectively attenuated these pro-apoptotic changes (Figure 5A,B). These findings demonstrate that liproxstatin-1 confers protection against H_2_O_2_-induced apoptosis in SH-SY5Y cells, likely through modulating the expression and activity of Bcl-2 family proteins and downstream caspase 3 activation.

### 2.5. Liproxstatin-1 Preserves Akt/mTOR Signaling in H_2_O_2_-Exposed SH-SY5Y Cells

**Figure 6:**
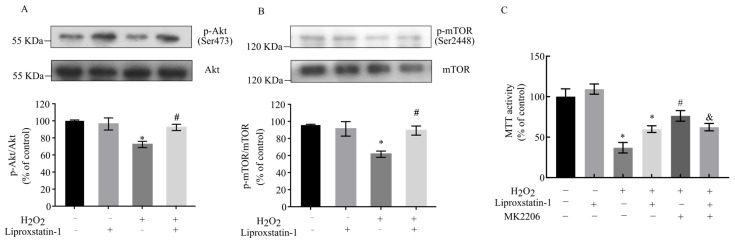
Liproxstatin-1 modulates Akt/mTOR signaling in H_2_O_2_-treated SH-SY5Y cells. SH-SY5Y cells were pretreated with 1 μmol/L liproxstatin-1 for 24 h prior to exposure to 500 μmol/L H_2_O_2_ for 2 h. (**A**) Total Akt and phospho-Akt (Ser473), (**B**) Total mTOR and phospho-mTOR (Ser2448) were determined by Western blot analyses. (**C**) Quantitative data are presented as mean ± SD from 3 (**A,B**) or 6 (**C**) independent experiments. * *p* < 0.05 versus control, # *p* < 0.05 versus H_2_O_2_-treated cells, & *p* < 0.05 versus cells co-treated with liproxstatin-1 and H_2_O_2_.

mTOR serves as a crucial autophagy regulator that integrates inputs from multiple signaling pathways, with primary regulation occurring through the PI3K/Akt pathway [12]. The Akt/mTOR axis additionally modulates essential cellular processes including mitochondrial biogenesis, dynamics (fusion and fission), mitophagy, and apoptosis [24]. To investigate whether liproxstatin-1 exerts mitophagy- and apoptosis-inhibitory effects via activation of the Akt/mTOR signaling in SH-SY5Y cells, we analyzed phosphorylation levels of Akt and mTOR. As demonstrated in Figure 6, H_2_O_2_ treatment significantly suppressed Akt and mTOR activation, while liproxstatin-1 pretreatment effectively attenuated these reductions. These findings suggest that liproxstatin-1 may modulate both mitophagy and apoptotic processes through Akt/mTOR pathway activation. The inhibitor of Akt, MK2206, attenuated the protective effect of liproxstatin-1 against H_2_O_2_-induced cytotoxicity (Figure 6C). These results indicate that liproxstatin-1 ameliorated H_2_O_2_-induced mitophagy and apoptosis via activating the Akt/mTOR signaling pathway in SH-SY5Y cells.

## 3. Discussion

Oxidative stress exerts immediate detrimental effects, including protein damage, DNA mutations, and increased membrane permeability, all of which contribute to the pathogenesis of neurodegenerative diseases [15]. Mitochondrial dysfunction is closely linked to an altered redox state [25]. ROS can permeabilize mitochondrial membranes, and the mitochondrial permeability transition facilitates the influx of protons and other molecules, leading to the loss of mitochondrial membrane potential and ATP depletion [25]. A decline in mitochondrial membrane potential, typically accompanied by reduced ATP levels, signifies impaired mitochondrial function [13]. In animal models, liproxstatin-1, a specific ferroptosis inhibitor, has been shown to preserve mitochondrial structural integrity, alleviate mitochondrial damage, and prevent oxidative stress [9,10]. In the present study, liproxstatin-1 similarly protected mitochondrial function and attenuated H_2_O_2_-induced cell death.

GSH serves as a reducing agent for glutathione peroxidases in the elimination of H_2_O_2_. The age-dependent decline in brain GSH levels has been associated with cognitive impairment in aging [26]. Elevated MDA concentration indicates oxidative stress and ROS generation [27]. Liproxstatin-1 has been demonstrated to suppress MDA level and restore GSH expression, suggesting that its antioxidant properties protect cells from H_2_O_2_-induced oxidative stress [11]. Consistently, in this study, liproxstatin-1 significantly attenuated the H_2_O_2_-induced depletion of GSH and accumulation of MDA in SH-SY5Y cells, thereby enhancing cellular antioxidant capacity and mitigating oxidative stress. Additionally, CAT plays a critical role in cellular defense against H_2_O_2_-mediated oxidative damage, and its upregulation reflects an adaptive response to oxidative stress [28]. The reduced CAT activity in the present study may be attributed to the antioxidative effects of liproxstatin-1, which helped maintain redox balance without requiring elevated CAT activity.

Apoptosis is a tightly regulated and evolutionarily conserved cell death process. Mitochondria serve as key mediators of apoptosis by releasing pro-apoptotic proteins into the cytoplasm [1]. Both autophagy and apoptosis can be triggered by exogenous H_2_O_2_ exposure [29]. In endplate chondrocytes, H_2_O_2_ stimulation induces mitochondrial dysfunction, elevated ROS production, and increased apoptosis rates, underscoring the central role of mitochondrial impairment in H_2_O_2_-induced apoptosis [30]. The pro-apoptotic Bcl-2 family protein Bax initiates programmed cell death by permeabilizing the outer mitochondrial membrane, thereby activating the caspase cascade. Conversely, anti-apoptotic Bcl-2 proteins inhibit Bax; an imbalance favoring pro-apoptotic signal leads to apoptosis [23]. Caspases are pivotal regulators of apoptotic pathways, with their activation representing a critical event in apoptosis [31]. For instance, chlorpyrifos exposure induces mitochondrial damage, upregulates cytochrome c, and triggers caspase 9 and caspase 3 activation, ultimately resulting in apoptosis [32]. In both oxidative death paradigms, liproxstatin-1 prevents cell death and rescues mitochondrial damage markers [33]. Consistent with these findings, our results demonstrate that liproxstatin-1 protects against H_2_O_2_-induced apoptosis by upregulating anti-apoptotic Bcl-2 and downregulating pro-apoptotic Bax.

Autophagy plays a crucial role in organelle quality control, with mitophagy representing the selective autophagic degradation of damaged mitochondria [30]. ROS act as signaling molecules that induce mitophagy, while oxidative stress exacerbates mitochondrial fragmentation, further amplifying ROS production [15]. The most well-characterized mitophagy pathway involves stabilization of the kinase PINK1 and recruitment of the ubiquitin ligase Parkin to impaired mitochondria [1]. Beclin 1 promotes autophagy, and its downregulation suppresses autophagic activity [19]. During autophagy, LC3-I is conjugated to phosphatidylethanolamine to form LC3-II, which integrates into autophagosomal membranes, making LC3-II a reliable marker for autophagy assessment [20]. In SH-SY5Y cells, H_2_O_2_ reduces cell viability, disrupts mitochondrial morphology, and induces mitophagy [12]. While mitophagy can eliminate ROS-damaged mitochondria to maintain homeostasis and promote cell survival, excessive mitophagy contributes to mitochondrial dysfunction and cell death [15]. In this study, liproxstatin-1 ameliorated the cytotoxicity of H_2_O_2_ through suppressing H_2_O_2_-induced mitophagy and significantly reduced PINK1/Parkin levels.

The PI3K/AKT/mTOR pathway regulates critical cellular processes, including DNA repair, proliferation, apoptosis, and inflammation [34]. mTORC1 inhibits autophagy by integrating upstream signals from PI3K and AKT [22]. Notably, AKT/mTOR signaling is activated in response to mitochondrial stress and can be modulated by Parkin [35]. For example, EphA2 overexpression inhibits autophagy by restoring H_2_O_2_-induced reductions in *p*-Akt and *p*-mTOR levels in SRA01/04 cells [36]. The PI3K/Akt/mTOR pathway also plays a neuroprotective role against oxidative stress [37]. Similarly, our findings suggest that liproxstatin-1 may inhibit H_2_O_2_-induced mitophagy and apoptosis via AKT/mTOR pathway activation.

Notably, liproxstatin-1’s neuroprotective effects should be contextualized within the broader landscape of existing agents targeting oxidative stress, mitophagy, or apoptosis, including natural products such as resveratrol and curcumin, synthetic antioxidants like N-acetylcysteine, and other ferroptosis inhibitors including ferrostatin-1 [38,39]. In contrast, liproxstatin-1 features ACSL4-mediated selective targeting of lipid peroxidation, enabling potent protection at low concentrations without disrupting redox homeostasis or inducing cytotoxicity; it converges on regulating excessive mitophagy, apoptosis, and Akt/mTOR pathway activation to address interconnected pathological cascades independently, without requiring synergistic combinations. Complemented by superior stability, lipid solubility, and blood–brain barrier penetration for CNS-targeted utility, as well as favorable safety profiles (≤1.5 μmol/L non-toxicity in SH-SY5Y cells, minimal in vivo toxicity) supporting long-term application, its mechanism of targeting oxidative stress-driven lipid peroxidation and dysregulated mitophagy also complements symptomatic therapies to potentially enhance combinatorial efficacy. These advantages, combined with our findings, highlight liproxstatin-1 as a targeted, safe, and effective neuroprotective candidate. However, these strengths must be interpreted alongside study limitations, including future studies directly comparing liproxstatin-1 with other neuroprotective agents in primary neurons or in vivo neurodegeneration models such as APP/PS1 mice for Alzheimer’s disease and MPTP-induced Parkinsonism are critical to further validate its therapeutic potential and translational value.

## 4. Materials and Methods

### 4.1. Materials

Fetal bovine serum (FBS), penicillin, streptomycin, Dulbecco’s modified Eagle’s medium (DMEM), trypsin, MitoTracker™ Green FM, LysoTracker™ Red, nonyl acridine orange (NAO) staining, and MitoSOX™ Red mitochondrial superoxide indicator were purchased from Thermo Fisher Scientific (Rockford, IL, USA). Liproxstatin-1 was purchased from MedChemExpress (Shanghai, China). 3-(4,5-dimethyl-2-thiazolyl)-2,5-diphenyl-2-H-tetrazolium bromide (MTT) and 2′,7′-dichlorodihydrofluorescein diacetate (DCFH-DA) were purchased from Sigma Chemical (St. Louis, MO, USA). MK2206, BCA protein assay kit, mitochondrial membrane potential assay kit, caspase 3 activity kit, ATP detection assay kit, Beyo ECL moon Western blotting detection system, HRP-labeled donkey anti-goat, goat anti-mouse, and goat anti-rabbit IgG (H + L) were purchased from Beyotime Institute of Biotechnology (Shanghai, China). The malondialdehyde (MDA), glutathione (GSH), and catalase (CAT) detection assay kits were purchased from Nanjing Jiancheng Bioengineering Institute (Nanjing, China). Antibodies for phosphorylated Akt (Ser473), Akt, phosphorylated mTOR (Thr2448), mTOR, LC3, and Beclin1 were purchased from Cell Signaling Technology (Danvers, MA, USA). Antibodies for PINK1, Parkin, and Tubulin were purchased from Santa Cruz Biotechnology (Dallas, TX, USA). Antibodies for Bcl-2 and Bax were purchased from Proteintech (Wuhan, China).

### 4.2. Cell Culture

Human neuroblastoma SH-SY5Y cells (obtained from the Shanghai Institutes for Biological Sciences, Chinese Academy of Sciences, Shanghai, China) were cultured in DMEM supplemented with 10% FBS and 1% penicillin-streptomycin. The cells were maintained at 37 °C in a humidified atmosphere containing 5% CO_2_ and 95% air.

According to preliminary concentration-screening assays and published literature [11,40], the concentrations of liproxstatin-1 used in subsequent experiments (0.5, 1, and 1.5 μmol/L) were selected.

### 4.3. MTT Assay

For cell viability measurements, cells (4 × 10^3^/well) were seeded in a 96-well culture plate, and the MTT assay was performed as described by Yan et al. [12]. Following treatment, cells were incubated with MTT at 37 °C for 4 h. The resulting formazan crystals were dissolved in DMSO, and the absorbance was measured at 570 nm using a microplate reader.

### 4.4. Measurement of ATP Content

Quantification of intracellular ATP content was performed using a commercially available ATP detection assay kit, following a modified protocol adapted from Cao et al. [10]. Briefly, post-treatment cells were lysed using the provided reagent and centrifuged at 12,000× *g* for 10 min at 4 °C to remove cellular debris. Supernatants or ATP standard dilutions were then mixed with the luciferin–luciferase reaction buffer. The enzymatic conversion of luciferin to oxyluciferin by luciferase generates bioluminescence proportional to the ATP concentration present in the sample. Luminescence signals were detected using a Synergy HTX Multi-mode Microplate Reader calibrated to photon-counting mode.

### 4.5. Measurement of Mitochondrial Membrane Potential

Mitochondrial membrane potential was evaluated using the JC-1 dye as previously described by Zhang et al. [6]. Following experimental treatments, cells were incubated with the JC-1 working solution for 20 min at 37 °C in a humidified atmosphere. After washing twice with warm PBS, cells were detached via trypsinization and resuspended in assay buffer. Fluorescence emission was measured using a fluorospectrophotometer at excitation/emission wavelengths of 490/525 nm (green fluorescence, monomeric JC-1) and 490/590 nm (red fluorescence, JC-1 aggregates). The relative mitochondrial membrane potential was calculated as the ratio of red to green fluorescence intensity.

### 4.6. Measurements of Total ROS and Mitochondrial ROS

The levels of total ROS were determined using DCFH-DA according to the methods of Xu et al. [41]. Briefly, cells (1 × 10^5^ cells/well) were seeded in 6-well plates. Post-treatment, cells were detached via trypsinization and incubated with 10 μmol/L DCFH-DA in serum-free medium for 30 min at 37 °C in the dark. Fluorescence intensity was measured using a microplate reader at excitation/emission wavelengths of 488/525 nm.

Mitochondrial superoxide production was assessed using MitoSOX™ Red and MitoTracker™ Green co-staining as previously described by Zhang et al. [42]. After treatment, cells were stained with MitoSOX™ Red mitochondrial superoxide indicator (5 μmol/L) diluted in DMEM supplemented with 10% FBS at 37 °C for 15 min, followed by three washes with PBS. Subsequently, cells were stained with MitoTracker™ Green (180 nmol/L) diluted in serum-free DMEM at 37 °C for 30 min. After washing with PBS, mitochondrial ROS production was assessed using an Olympus BX53 fluorescence microscope (excitation wavelength: 480–550 nm; emission wavelength: 590 nm). Colocalization analysis was conducted using ImageJ software (v 2.1.4.7).

### 4.7. Measurement of Caspase 3 Activity

Caspase-3 activity was measured using a colorimetric assay kit following the manufacturer’s instructions and the methods of Xu et al. with minor modifications [41]. After treatment, cells were lysed using the provided buffer, and protein concentrations were standardized. Lysates were incubated with Ac-DEVD-pNA substrate in reaction buffer for 10 h at 37 °C. pNA release was quantified at 405 nm using a spectrophotometer (Yuanxi, Shanghai, China).

### 4.8. Measurement of MDA

The MDA content was measured using the thiobarbituric acid assay as previously described by Jindagul et al. with minor modifications [40]. After treatment, cells were harvested by trypsinization and lysed using an ultrasonic cell crusher. Subsequently, testing reagents were added according to the manufacturer’s instructions. Samples were boiled for 40 min in the dark, cooled on ice, and centrifuged at 10,000× *g* for 10 min. Absorbance was measured at 532 nm.

### 4.9. Measurement of GSH

Intracellular GSH levels were determined using a commercial enzymatic recycling assay according to the manufacturer’s instructions and the methods of Cha et al. with minor modifications [43]. After treatment, cells were harvested by trypsinization and lysed using an ultrasonic cell crusher. The supernatant was collected and mixed with the testing reagents. The absorbance at 405 nm was monitored using a microplate reader.

### 4.10. Measurement of CAT

CAT activity was quantified using the ammonium molybdate method, which terminates CAT-mediated H_2_O_2_ decomposition by forming a yellow complex with remaining H_2_O_2_, according to the manufacturer’s instructions and the methods of Yang et al. with minor modifications [44]. After treatment, cells were lysed via ultrasonic disruption. Subsequently, the testing reagents were added to the supernatant. Absorbance was measured at 405 nm using a microplate reader.

### 4.11. Mitochondria and Lysosomes Colocalization

Mitophagy levels were assessed via colocalization of MitoTracker™ Green and LysoTracker™ Red as described by Feng et al. with minor modifications [16]. Cells (2 × 10^5^/well) were seeded in a 6 cm plate. After treatment, cells were stained with Lyso-Tracker™ Red for 15 min at 37 °C, fixed with 4% paraformaldehyde for 15 min, and subsequently stained with MitoTracker™ Green in serum-free medium for 30 min at 37 °C. Fluorescence images were captured using an Olympus BX53 fluorescence microscope at 490/516 nm (MitoTracker™ Green) and 577/590 nm (LysoTracker™ Red). Manders overlap coefficients were calculated for 30–35 randomly selected cells per group using Image Pro Plus 6.0 software with the Formula (1), where S1*_i_* and S2*_i_* represent the signal intensity of individual pixels in channel 1 (red) and 2 (green), respectively (*_i_* represents a single pixel) [45]. The values from 3 parallel treatments were then averaged.

(1) $$\frac{\sum_{i}{S1}_{i}\times {S2}_{i}}{\sqrt{\sum_{i}{\left({S1}_{i}\right)}^{2}\times \sum_{i}{\left({S2}_{i}\right)}^{2}}}$$

### 4.12. Analyses of Mitochondrial Mass

The mitochondrial mass was determined using the fluorescent dye NAO, a fluorescent dye that selectively binds to cardiolipin in the inner mitochondrial membrane. After treatment, cells were incubated with 50 nmol/L of NAO at 37 °C for 30 min as described by Galber et al. [18]. Fluorescence intensity was measured using a Synergy HTX microplate reader (excitation 488 nm, emission 533 nm).

### 4.13. Western Blot Assays

Whole-cell lysates were prepared using cell lysis buffer after treatment, following the protocol of Yan et al. [12]. Samples were sonicated on ice for 2 min and centrifuged at 12,000× *g* for 10 min at 4 °C. Protein concentration was determined via BCA assay. Equal amounts of protein (10 μg) were separated by 10% SDS-PAGE and transferred to PVDF membranes. Membranes were blocked with 5% BSA in TBST and probed with primary antibodies overnight at 4 °C, followed by HRP-conjugated secondary antibodies for 1 h at room temperature. Protein bands were visualized using Beyo ECL Moon chemiluminescence substrate and quantified via densitometry using ImageJ software.

### 4.14. Statistical Analysis

Quantitative data were analyzed using GraphPad Prism 7.00. Statistical analyses were performed using two-tailed Student’s t-tests, and a *p*-value of less than 0.05 (*p* < 0.05) was considered statistically significant.

## 5. Conclusions

In summary, this study demonstrates that liproxstatin-1 attenuates H_2_O_2_-induced cytotoxicity by preserving mitochondrial function and enhancing cell survival. Specifically, liproxstatin-1 reduces H_2_O_2_-induced PINK1/Parkin-mediated excessive mitophagy and apoptosis via activating the Akt/mTOR signaling pathway. These findings suggest that liproxstatin-1 exerts protective effects against oxidative stress, mitophagy, and apoptosis, highlighting its potential as a therapeutic agent for neuronal injury.

## Data Availability

The raw data supporting the conclusions of this article will be made available by the authors on request.

## Abbreviations

AD, Alzheimer’s disease; Akt, protein kinase B; CAT, catalase; DCFH-DA, 2’,7’-dichlorodihydrofluorescein diacetate; DMEM, Dulbecco’s modified Eagle’s medium; FBS, Fetal bovine serum; GSH, glutathione; MDA, malondialdehyde; mTOR, mammalian target of rapamycin; MTT, 3-(4,5-dimethyl-2-thiazolyl)-2,5-diphenyl-2-H-tetrazolium bromide; NAO, nonyl acridine orange; PD, Parkinson’s disease; ROS, reactive oxygen species.
